# Rehabilitation and management outcomes of musculoskeletal injuries in a major referral hospital in Ghana

**DOI:** 10.1186/s12891-019-2423-5

**Published:** 2019-01-24

**Authors:** Eric Lawer Torgbenu, Evans Yayra Kwaku Ashigbi, Maxwell Peprah Opoku, Sandra Banini, Emmanuel Barima Agyemang Prempeh

**Affiliations:** 1 Department of Physiotherapy and Rehabilitation Sciences, University of Health and Allied Sciences, School of Allied Health Sciences, Ho, Ghana; 2 Faculty of Education, University of Tasmania, Launceston, Australia; 3 Department of Physiotherapy, Volta Regional Hospital, Ho, Ghana; 4 Teaching and Examinations Unit, University of Health and Allied Sciences, Ho, Ghana

**Keywords:** Musculoskeletal injuries, Epidemiology, Pain management, Diagnosis, Retrospective studies, Ghana

## Abstract

**Background:**

The devastating impact of musculoskeletal injury (MSI) on human lives, the economy, and health services cannot be overemphasised. This has ignited discussion at international fora, as countries have been exhorted to prioritise management of MSI in order to maintain a healthy society. In the Ghanaian context, the knowledge base management of MSI is very low, which has provided the impetus to explore the management of MSI and the rehabilitation systems at a tertiary hospital in Ghana.

**Methods:**

The study was a retrospective cross-sectional study, using the consecutive sampling method to recruit patients who were discharged after admission at the accident and emergency unit, as well as patients undergoing orthopaedic review, at the St. Joseph’s Orthopaedic Hospital in Koforidua over a six-month period.

**Results:**

A total of 269 musculoskeletal injury patients were recruited for the study. Half of the participants (51%) had had surgery in addition to pain medication. The overall mean recovery days were 26.81 ± 33.94 days, and the average disability days spent in the hospital were estimated at 16.54 ± 27.97 days. Individuals reported financial constraints as a major challenge to their full participation in rehabilitation.

**Conclusion:**

The findings of this study have implications for policymaking in Ghana. Particularly, the need to improve health facilities to enable MSI patients to seek treatment is highlighted. Also, the need to train health professionals who will be able to administer appropriate medication for MSI patients is discussed extensively.

## Background

Musculoskeletal injury (MSI) refers to damage to the muscular or skeletal system, which is usually due to strenuous activity [1, 2]. MSI can affect the muscles, nerves, tendons, ligaments, joints, blood vessels, neck or lower back [3]. Globally, a significant proportion of the population is at risk of acquiring MSI. For instance, between 1990 to 2015, it has been estimated that over 17.8 million people were diagnosed with MSI in the United Kingdom (UK) [4], and the prevalence in developing countries is increasing. Consequently, in 2000, the WHO launched the Bone and Joint Decade 2000–2010, to raise awareness of the increasing societal impact of MSI and disorders [5–7]. The campaign raised awareness of the effects of chronic non-communicable diseases such as MSI, and it exhorted countries to develop systems to reduce the burden of these diseases [5]. Unfortunately, despite the high prevalence of MSI in developing countries, there is little epidemiological data about management and the burden of MSI [8–10]. While there is considerable funding for control of communicable diseases, little attention has been paid to documentation, prevention or management of MSI in developing countries, such as Ghana [11, 12]. There is therefore a need to establish baseline information about the management of MSI in a tertiary hospital in Ghana, which can inform policy direction in healthcare delivery.

The negative impact of MSI on human lives, productivity, and financial resources cannot be overemphasised. For instance, MSI contributes to a sizable burden on medical resources, as considerable funds are needed annually to support hospitals, and even patients [13]. In the US, annual injury-attributable medical expenditure is consistently around $200 billion [13, 14]. Due to the scarcity of financial resources to provide infrastructure in health facilities, it is possible that developing countries may be facing challenges with respect to treating MSI. In fact, anecdotal evidence shows that in developing countries health systems and individuals with MSI are facing challenges, and this has had an adverse impact on disease management and rehabilitation. These challenges include the high cost of treatment, the lack of health facilities, the long distances to reach facilities, poverty, the inadequacy of surgeons, and inability to transport patients to hospitals, as a result of poor transportation systems [15, 16]. These systemic challenges need attention in efforts at promoting a healthy population which will contribute substantially to national development. At the individual level, MSI leads to pains, discomfort, and dissatisfaction among individuals, depression, anxiety, and psychological problems, resulting in poor work outcomes [17–19]. It is unsurprising therefore that available evidence shows that individuals with MSI always record lower scores on the Quality of Life Scale [19, 20]. Additionally, Larsson and Nordholm [21] as well as Picavet and Hoeymans [18] also found that MSI results in limited participation of patients in social and health activities, such as attending physical activity programmes and activities that require movement. It is therefore imperative that MSI patients are promptly taken care of, to prevent prolonged disability and worsening conditions.

Adopting an evidence-based approach to MSI management in a context will help to understand the management trajectory, which will provide useful guidelines to practitioners [22–24]. However, there is a lack of consensus in the literature regarding the management of MSI conditions. While some studies have found pharmacological treatment to be effective [25], other studies have reported the usefulness of non-pharmacological treatments for MSI [24]. For example, according to Van der Roer, De Lange, Bakker, de Vet, and Van Tulder [26], management of MSI, with the emphasis on traumatic fractures, remains a controversy. They further opined that individuals with injuries emanating from trauma could benefit from both operative and conservative treatment. Although there is insufficient literature on the management of traumatic fracture, surgical intervention is mainly used at the discretion of the consulting surgeon [27]. Additionally, Van Tulder, Malmivaara and Koes [28] also identified exercise, topical applications, and oral painkillers and injections with corticosteroids to be effective in the management of MSI. Additionally, physiotherapy is another intervention which is used in the management of muscular pain [29]. This process involves the use of exercise, with or without mobilisation, enabling short-term recovery and long-term improvement in function [30]. Due to the different approaches to managing MSI, and seeming disparities in patients’ satisfaction [31], it is important that context-specific cases are studied, in order to understand the treatment patterns in a major orthopaedic hospital and to offer policy directions. Therefore, this study sought to answer the following questions: (1) What are the treatments administered to MSI patients in a tertiary hospital in Ghana? (2) What are the quality of life and the length of hospital stay for patients receiving treatment for MSI in a tertiary hospital in Ghana? and (3) What are the factors affecting MSI patients in terms of assessing treatment and rehabilitation?

## Methods

### Study participants

This study forms part of a larger study which examined management outcomes of MSI in a tertiary hospital in Ghana [32]. The researchers used the consecutive sampling technique to recruit participants for the study. Lunsford and Lunsford [33] describe consecutive sampling as drawing on available population for data collection and there is likelihood for people from diverse backgrounds to be recruited. All patients receiving treatment for MSI at the hospital were eligible for participation. With this, the researchers included all accessible subjects who met the inclusion criteria as and when they arrived at St. Joseph’s Orthopaedic Hospital (the accident and emergency unit, as well as the orthopaedic wards).

### Study design

The study was a retrospective cross-sectional design involving MSI patients [34] receiving orthopaedic care at St. Joseph’s Orthopaedic Hospital, Koforidua, Ghana. The hospital was built by the Catholic Mission whose core mandate is to make orthopaedic treatment accessible to all persons at a cheaper cost [35]. The facility has about 180 beds and serves as a major referral centre for rehabilitation in Ghana and neighbouring West African countries such as Burkina Faso, Ivory Coast, Liberia, Nigeria, and Togo [36]. St. Joseph’s Hospital sees over 1000 surgical cases per year, with nearly 60% being orthopaedic trauma-related [37].

### Instrument

The questionnaire used in the data collection was developed by the researchers from a review of the literature [33, 38–40]. The questionnaire used to collect data was made up of six parts: demographic characteristics, the causes of the musculoskeletal injuries, the severity of the injuries, the management received for various injuries, the Oswestry Disability Index, and accessibility of rehabilitation services.

The demographic data collected included: gender, level of education, age, marital status and home region. In relation to causes of MSI, severity and management, participants were asked to circle an option that best explain their condition.

Also, a modified version of the Oswestry Disability Index was adapted for this study to gather information on the quality of life of the participants. The sections of the questionnaire included questions to estimate the physical functioning level of the participants [41], and it was adapted to suit all participants with musculoskeletal injury. The questionnaire subjectively assessed items including pain intensity of the individuals, personal care, and activities of daily living, including lifting, walking, sitting, standing, and sleeping. It also assessed the participants’ sex life, their social life, and their travelling. The scale is graded from 0 to 100, where the range from 0 to 20% represents maximum quality of life or maximum physical functioning level, 21 to 40% represents moderate disability, 41 to 60% represents severe disability, 61 to 80% signifies “crippled”, and 81 to 100% signifies “bedridden or no physical functioning”. The internal consistency of the Oswestry Disability Index was .69. The duration of disability days was also estimated from the number of days an individual stayed on admission before discharge. This was calculated as the length of hospital stay. The mean recovery period was estimated from the total duration of treatment the individual received for the condition. This involved the estimated disability days and the number of days an individual attended the outpatient orthopaedic reviews. The conditions of participants were only inferred from the patients’ folders to confirm the diagnosis before discharge.

The last part of the instrument was accessibility of rehabilitation services which was made up of closed and opened ended questions. The questionnaire was given to academics in two universities for their contributions. The instrument was then piloted to ensure that it met the necessary standards of scientific research. Various parts of the tool were modified before it was used for the data collection. The computed internal consistency and Cronbach’s alpha for the questionnaire was .72.

### Procedures

This study was conducted from November 2013 to April 2014. The study and its protocols were approved by the Human Ethics Research Committee at the School of Medical Sciences, Kwame Nkrumah University of Science and Technology, the Ghana Health Service, and administrators at the health facility where this study was conducted. After the necessary approval was granted, notices were posted at various departments to inform health professionals about the study and their support during the data collections. The data were collected on Thursdays because that was when most patients had appointment for review and consultations. After treatment, health professionals asked patients if they were willing to participate in this study. Patients who were willing to participate were then referred to the research assistant responsible for the data collection. The objectives of the study and its relevance were explained to prospective participants who signed informed consent before completing the questionnaires. While some participants completed the questionnaires themselves, others were assisted by the research assistant. Specifically, the research assistant translated the questions to Twi language which is a local language used by the people. Parents or caregivers of minors were made to assent for them before they participated in the study. Participation was voluntary and patients were not rewarded to participate in the study. Participants were free to withdraw from the study without any consequences and the duration for completion of the questionnaire ranged from 45 min to an hour.

### Data management and analysis

The first author checked all questionnaires for completeness before entry into Statistical Package for Social Science, version 20, that was used for the data analysis. The assumptions underlying the inferential statistics were violated. Thus, the research team decided to report descriptive statistics. To answer research question 1, 2, and 3, we calculated means, standard deviations, modes and medians. More so, the means and standard deviations were illustrated using tables, to present the levels of MSI, causes, and management received for the various MSI. Additionally, to answer research question 4, the open-ended responses were subjected to frequency counts to get percentage of participants who encountered challenges.

## Results

### Demographic characteristics of the participants

Within the six-month period, a total of 320 questionnaires were distributed, and 269 were returned, giving a response rate of 84%. The ages of the respondents ranged from 1 to 82 years. Male participants constituted 137 (51%) of the participants, while female patients were 132 (49%). A few of the participants (5%) were children under 5 years [see 32].

### Treatment

Overall, a total of 157 participants reported having injury, while 112 reported no injury, and were thus excluded from further analysis. Table 1 presents the distribution, with percentages of different types of treatment received for various musculoskeletal injuries. All the participants who received surgery also had pain medication. Surgery was performed on over half of the participants (51%), while 38% of the participants were given only pain medication. About 5% of participants indicated that they received other forms of treatment. Participants with fracture cases received surgery and pain medication. More surgeries were performed for fracture cases (32%) than for any other condition.

**Table 1:** Distribution of treatment received by respondents

Treatment Received	Causes	Frequency	Percentages (%)
Surgery and pain medication			
	Fracture	51	32
	Arthritis	9	6
	Dislocation	8	5
	Back pain	0	–
	Ligament injury	8	5
	Bone infection	1	1
	Amputation	3	2
	Tendon injury	0	–
	Total	80	51
Physiotherapy			
	Fracture	3	2
	Arthritis	1	1
	Dislocation	0	–
	Back pain	1	1
	Ligament injury	1	1
	Bone infection	0	–
	Amputation	0	–
	Tendon injury	5	3
	Total	11	7
Pain Medication			
	Fracture	18	11
	Arthritis	7	4
	Dislocation	6	4
	Back pain	22	14
	Ligament injury	0	–
	Bone infection	1	1
	Amputation	0	–
	Tendon injury	6	4
	Total	60	38
Others			
	Fracture	3	2
	Arthritis	0	–
	Dislocation	1	1
	Back pain	2	1
	Ligament injury	0	–
	Bone infection	0	–
	Amputation	0	–
	Tendon injury	0	–
	Total	6	4

### The duration of rehabilitation

Table 2 presents the duration of treatment received by respondents. This was calculated from respondents’ folders, which contained information on the number of days they had stayed at the hospital. One-hundred-and-thirty-eight participants (88%) indicated that a rehabilitation programme was designed for them after their first line management. Of this number, 90 participants (66%) confirmed that they went through all the stages of the programme, while the remaining 48 responded otherwise.

**Table 2:** Distribution of mean recovery period and mean disability days

Characteristics	Mean recovery period in days (SD)	Mean disability days (SD)
Fracture	29 (34)	16 (17)
Arthritis	26 (42)	9 (9)
Dislocation	17 (11)	19 (27)
Back pain	29 (36)	24 (48)
Ligament injury	31 (35)	12 (6)
Amputation	72 (68)	12
Tendon injury	13 (7)	10 (7)
Overall	27 (34)	17 (28)

*SD* Standard Deviation

The overall mean disability days of the participants was recorded to be 17 ± 28 days, and the mean duration of treatment received by the participants was 27 ± 34 days. Amputation recorded the longest mean recovery period 72 ± 68, while tendon injury recorded the least recovery days (7–13 days). Regarding average disability days, back pain recorded the most mean disability days (24–48 days), while arthritis recorded the least mean disability days (9 ± 9).

### Quality of life of participants

The Oswestry Disability Index was used to gather information on quality of life. Table 3 demonstrates the quality of life of participants as per their MSI condition. Overall, all the participants who reported to the rehabilitation unit had conditions that ranged from minimal to almost bedridden. The total mean physical functioning level of the participants in the study was 35–38. Participants who suffered back pain recorded the highest mean functioning levels (79 ± 15), while participants with bone infection recorded lower mean physical functioning levels. More than half of the population (72) suffered severe disability, recording 80–100% on the scale, representing total dependency and possibly requiring much assistance to be able to carry out their daily living activities.

**Table 3:** Distribution of mean physical functioning of participants

Physical functioning	Fracture	Arthritis	Dislocation	Back pain	Ligament injury	Bone infection	Amputation	Tendon injury	Total
0–20%	7	1	1	12	0	0	0	5	26
21–40%	12	2	3	9	0	0	0	2	28
41–60%	5	0	1	3	0	0	0	2	11
61–80%	11	5	1	1	2	0	0	0	20
81–100%	40	9	9	0	8	2	2	2	72
TOTAL	75	17	15	25	10	2	3	11	157
Mean	31	28	31	79	8	–	22	62	38
QOL (SD)	32	28	32	15	10		29	38	35

*QOL* quality of life

### Challenges to accessing rehabilitation services

On average, respondents attended rehabilitation services daily, weekly, occasionally, and once only. The results indicate that over 60% of the participants accessed rehabilitation services weekly, while less than 2% of the respondents accessed these services occasionally. Also, of the 138 patients who were referred to have rehabilitation services as per their condition, only 85 (62%) responded to the questions regarding “how accessible they found the hospital”, 87 (63%) responded to the questions regarding the challenges faced with the rehabilitation, and 88 (64%) responded to the questions regarding the frequency of attending rehabilitation services. Of the number who responded to how accessible the hospital was to them, 21% indicated that the hospital was not accessible to them, 68% responded that the hospital was accessible, and the remaining 8% said the hospital was very accessible to them. In addition, the respondents who went for rehabilitation services identified challenges such as financial constraints, lack of time, lack of commitment, and others. Of the number who responded, 70% identified that financial constraints were a major challenge, while 14% responded that there were other challenges, but they were not specific about the challenges.

## Discussion

Treatment of MSI is a challenge for healthcare professionals, which could be mitigated by availability of data to guide practice. This calls for more attention to be directed towards measures that are aimed at identifying the patterns of management and making them available to health professionals. Particularly, getting the initial procedure wrong has consequences for individuals, who may spend a long time in bed, which may affect jobs, dependants, or the economy in general [42]. This informed the need to conduct this study at a major referral hospital that serves Ghana and its neighbouring countries, to document the patterns in the management of MSI and rehabilitation services. This study attempted to gather evidence of the management of MSI, which may inform health practices nationally, as well as in countries whose health systems are similar to that of Ghana.

Key findings that emerged in this study were the use of pain medication and surgery for treatment of MSI. Without question, the pain associated with MSI has contributed to the use of pain medication by participants [26]. However, in relation to surgery, it is possible that the health facility where this study was conducted has many surgeons who are readily available, and there are thus a high number of surgeries. These findings are partly consistent with previous studies, which have found that surgery and pain medication are ways to treat MSI [1]. However, the findings are inconsistent with previous studies, in that they reported exercise as an alternative method of treatment [28]. Apparently, the high number of surgeries could also be attributed to the severity of conditions reported at the hospital. Perhaps as a result of challenges in accessing MSI treatment [16], patients got to the hospital when the only option was to perform surgery. Notwithstanding, this finding suggests the need for health systems to be well equipped and stocked with health professionals who will be able to perform surgery or administer appropriate medication to patients. Particularly, as early intervention has been found to be critical in treatment of MSI, patients will receive appropriate treatment once systems have been resourced to provide quality health services.

Many of the participants suffered severe disabilities, with those with bone infections having lower quality of life. This finding is partly consistent with previous studies, which have reported poor quality of life among individuals diagnosed with MSI [17–20]. This result could be attributed to lack of facilities and health professionals at facilities to meet the increasing number of patients with MSI. The health facility could be inundated with increasing cases of MSI, thus making it difficult for it to effectively support all patients. This trend should be a wake-up call to health policymakers, because of the fact that individuals with disabilities are not tolerated in Ghana [43, 44]. Worst of all, they are unable to find jobs, and they live in deplorable living conditions [44–46]. Therefore, it is crucial for the government to resource health facilities to avert this situation.

The disabling effect of participants’ condition and the many days required for treatment were reported. The findings showed that a number of participants spent many days in the hospital, which suggests the disabling effect of the condition. Participants who had amputations reported the longest mean recovery period. This was not surprising, as individuals who have had amputations will need more time to recover and be able to cope with new and unfamiliar environments [47]. It is possible that many individuals will have to cope with the use of assistive devices, such as crutches, ankle foot orthoses, and transfemoral and transtibial prostheses, and they will require quite some duration time for rehabilitation to enable them to familiarise themselves with their new ambulatory methods [48, 49].

Rehabilitation is crucial in our modern society when it comes to integrating people into the world of work and promoting independent living in the event of physical injuries [6]. It is for this reason that the WHO, through its Bone and Joint Decade (2000–2010), is encouraging countries to put measures in place and develop their rehabilitation facilities [50]. In this study, only a few participants went for rehabilitation, despite being referred by health professionals. This was inferred from the number of participants who reported to have received some form of rehabilitation. Previous studies in developing countries have found that MSI patients were unable to receive treatment because of limited facilities, poverty, and having to cover long distances to seek healthcare [15]. In Ghana, there is a high rate of poverty, which is a barrier to vulnerable groups in terms of accessing healthcare and services [51–54]. This may discourage participants from accessing rehabilitation facilities. The wealth of every nation is a healthy population who will contribute substantially to the economic development of the country [55]. Therefore, developing countries have to increase and expand rehabilitation facilities to make the services accessible to all people. Specifically, the government and its healthcare agencies need to work at making rehabilitation services accessible to all, regardless of place of residence or status within society.

The participants acknowledged that financial problems and time spent accessing treatment are major challenges that they face. This finding confirms previous studies, which have reported poverty and the high cost of seeking MSI treatment as challenges [15, 16]. It is possible that since the health facility is one of the specialised centres providing orthopaedic services in Ghana and beyond, there might be many patients seeking care at the facility. At such a specialised facility, the cost of treatment of MSI could be high, and probably middle-income earners could afford such treatment. Since most inhabitants in Ghana, and especially in the area where the hospital is situated, are poor farmers and traders [56], it is expected that their major complaint will be financial constraints. Ordinary people in Ghana might not be able to afford services in hospitals should they develop MSI. Therefore, it is required that further research be carried out on a large scale to examine the average treatment cost of MSI in Ghana. This will assist organisations and the government in planning policies to help waive some of the costs of MSI treatment, so as to enhance its accessibility.

## Conclusion

MSI is a major public health problem, due to its negative impact on individuals and society and the national economy [57]. This study attempted to document the management patterns of MSI in Ghana, so as to develop baseline information which will be relevant to policymakers. The major treatments administered for study participants were surgery, physiotherapy, and pain medication. Amputation was found to have the longest mean recovery period, while tendon injury had the shortest mean recovery period. Many participants had lower quality of life, due to the disabling effect of their condition. Moreover, financial barriers and long hospital stays were barriers reported by many participants. The results appear to underscore the need for Ghana to put mechanisms in place to prioritise treatment of MSI in Ghana.

The results of this study could have implications for policymaking in Ghana. Particularly, the results underscore the need for Ghana to resource other health facilities, as well as improve infrastructure at the study site, in order to enhance MSI treatments. Also, due to the high cost of treatment to participants, the government could consider extending the National Health Insurance Scheme premiums to cover rehabilitation services in various healthcare institutions. This will plausibly remove poverty barriers that impede healthcare access. There is also a need to establish community-based rehabilitation and community follow-up models to address referral systems and offer social and vocational support systems to MSI patients. This will enhance individuals’ integration into the community and will probably lessen the disabling effect of their condition.

It is important to point out here that the study has a number of limitations, which may affect generalisability of the study results. The current research considered the major treatment received, without necessarily taking into consideration the fact that participants might have received multiple treatments for their condition. Future studies could take this into consideration. Also, we cannot tell the effectiveness of treatment given to participants who took part in this study. Future studies should follow up to establish the effectiveness of treatment procedures patients may have received. Although this current study was limited to one health facility, it should be noted that the outcome of the study could be a reflection of the situation in other parts of Ghana, as the protocols and modalities involved in the management of MSI are the same across the country.

## Abbreviations

AIS: Abbreviated Injury Scale
LMIC: Low and Middle-Income Countries
MSI: Musculoskeletal Injury
QOL: Quality of Life
UK: United Kingdom
WHO: World Health Organization
